# Impact of non-dialysis chronic kidney disease on survival in patients with septic shock

**DOI:** 10.1186/1471-2369-14-77

**Published:** 2013-04-02

**Authors:** Julien Maizel, Romain Deransy, Benedicte Dehedin, Edouard Secq, Elie Zogheib, Elisabeth Lewandowski, Chritstophe Tribouilloy, Ziad A Massy, Gabriel Choukroun, Michel Slama

**Affiliations:** 1 Medical Intensive Care Unit, Department of Nephrology, Amiens University Medical Center, Amiens, France and INSERM U-1088, Jules Verne University of Picardie, Amiens, France; 2 Department of Anesthesiology and Intensive Care, Amiens University Medical Center, Amiens, France and INSERM U-1088, Jules Verne University of Picardie, Amiens, France; 3 Department of Medical information, Amiens University Medical Center, Amiens, France; 4 Department of Cardiology, Amiens University Medical Center, Amiens, France and INSERM U-1088, Jules Verne University of Picardie, Amiens, France

## Abstract

**Background:**

Chronic kidney disease (CKD) is known to expose the patient to a high risk of death due to cardiovascular and infective causes. In parallel, septic shock is a major challenge for cardiovascular and immune system. Therefore we tried to determine whether non-dialysis CKD, defined as a baseline estimated glomerular filtration rate (eGFR) <60ml/min/1.73m^2^, for three months prior to the onset of septic shock is an independent risk factor for death.

**Methods:**

All patients treated in a teaching hospital medical ICU for septic shock between January 2007 and December 2009 were retrospectively analyzed. Patients in whom baseline eGFR could not be determined (n=14) or patients treated by chronic dialysis (n=21) or kidney transplantation (n=14) were excluded. A total of 163 patients were included. The population was divided according to baseline eGFR ≥ 60ml/min/1.73m^2^ (non-CKD group, n=107) and < 60ml/min/1.73m^2^ (CKD group, n=56). Twenty-eight-day and 1-year survival curves were plotted. Prognostic factors were determined using Cox proportional hazards models.

**Results:**

Baseline eGFR was significantly higher in the non-CKD group than in the CKD group (81 (67–108) vs. 36 (28–44) ml/min/1.73m^2^, respectively; p=0.001). Age, SAPS II, serum creatinine on admission and the number of patients with a history of diabetes, hypertension, heart failure, peripheral artery disease, coronary artery disease and statin medication were significantly higher in the CKD group than in the non-CKD group. The mortality rate was lower in the non-CKD group than in the CKD group after 28 days (50% vs. 70%, respectively; p=0.03) and 1 year (64% vs. 82%, respectively; p=0.03). On multivariate analysis, the dichotomous variable CKD (eGFR < 60ml/min/1.73m^2^) remained significantly associated with the 28-day and 1-year mortality.

**Conclusions:**

Non-dialysis CKD appears to be an independent risk factor for death after septic shock.

## Background

Septic shock is a major therapeutic problem in intensive care units (ICUs) and constitutes a severe hemodynamic and immune challenge for the patient. The high incidence and high mortality rate associated to septic shock resulted in an economic burden of nearly $17 billion per year during the 90’s in the United States [1,2]. More recently the average per-case cost for hospitalization due to severe sepsis in ICU was estimated between 29,990 and 36,218$ [3].

Chronic kidney disease (CKD, defined by an estimated glomerular filtration rate (eGFR) < 60ml/min/1.73m^2^ stable for more than three months) is estimated to affect over 8 million people in the United States and these patients are exposed to increased risks of death, cardiovascular events and hospitalization [4,5]. Moreover, CKD is also an independent risk factor for death due to lung or bloodstream infections [6,7]. Given that (i) CKD exposes the patient to a high risk of death due to cardiovascular and infective causes and (ii) septic shock is a major immune and hemodynamic challenge, we decided to study whether CKD prior to onset of septic shock is an independent risk factor for death.

## Methods

### Study population

We retrospectively analyzed all consecutive incident patients treated for septic shock in the medical ICU of our University medical center (Amiens, France) between January 1st, 2007, and December 31st, 2009. Septic shock was defined according to the international consensus statement [8]. If patients experienced several episodes of septic shock during hospitalization, only the first episode was analyzed. Patients were then classified according to their baseline eGFR (defined as the eGFR during the 3 months preceding septic shock). Baseline eGFR was calculated using the four-variable Modification of Diet in Renal Disease (MDRD) equation [5]. Patients were included in the non-CKD group when they had a history of stable eGFR (measured under stable conditions, e.g. during 2 scheduled consultations) ≥ 60ml/min/1.73m^2^ during the 3 months preceding the onset of septic shock. When this previous eGFR was not available, the eGFR value after recovery from septic shock was used and, when it was ≥ 60ml/min/1.73m^2^ (in the absence of renal support), these patients were included in the non-CKD group (n=32) assuming this value to correspond to eGFR before the onset of septic shock. Patients were included in the CKD group when they presented a stable baseline eGFR < 60ml/min/1.73m^2^ for 3 months preceding the onset of septic shock.

Patients lacking longitudinal eGFR data and with an eGFR < 60ml/min/1.73m^2^ after recovery from septic shock were excluded from this study (n=14), as it was impossible to determine whether or not the low post-shock eGFR reflected their previous condition. Patients on chronic hemodialysis or peritoneal dialysis (n=21) and those who had undergone a kidney transplantation (n=14) were also excluded from this study.

All patients were treated according to the international guidelines for management of severe sepsis and septic shock, except that (i) echocardiographic parameters (instead of central venous pressure) were used to assess fluid responsiveness and (ii) recombinant human activated protein C was not administered [8].

In accordance with French legislation, the local institutional review board (CPPNord-Ouest II, Amiens University hospital, France) approved the study protocol.

### Data collection

The following demographic data were collected for each patient: age at admission, gender, history of diabetes, hypertension, heart failure, peripheral artery disease, coronary artery disease, COPD, statin administration, admission for surgery or other reasons and infection sites. Plasma hemoglobin and creatinine levels on admission were also recorded. The Simplified Acute Physiology Score II (SAPS II) was recorded within 24 hours of admission.

Onset of septic shock was considered to correspond to administration of catecholamine. Non-ICU-acquired septic shock was defined as septic shock present on ICU admission or occurring within 48 hours of admission. Acquired septic shock was defined as shock occurring more than 48 hours after ICU admission. The number of days without catecholamines or mechanical ventilation was calculated by subtracting the number of days with catecholamines or mechanical ventilation from 28 days or the number of days until death or ICU discharge (if the latter occurred within 28 days of admission) [9]. The length of stay was defined as the number of days between ICU admission and discharge or death. All patients were classified according to the “Risk, Injury, Failure, Loss, End-stage” (RIFLE) acute kidney injury criteria by taking into account diuresis and plasma creatinine level during the first 24 hours of septic shock [10].

Twenty-eight-day and one-year mortality rates after onset of septic shock were calculated.

### Statistical analysis

Data are expressed as median and interquartile range (IQR). Proportions were compared by a Chi-square test and quantitative variable were compared by a Mann–Whitney test.

Patient survival curves were plotted according to the Kaplan–Meier method and were compared in a Cox–Mantel log rank test.

A Cox proportional hazards model was used to identify baseline prognostic factors associated with 28-day and 1 year mortality. Univariate analysis initially tested all baseline variables: age, gender, history of diabetes, hypertension, heart failure, peripheral artery disease, coronary artery disease, COPD, statin use, baseline eGFR, CKD status (baseline eGFR < 60ml/min/1.73m^2^; yes or no), creatinine on admission, hemoglobin (Hb) on admission and SAPS II. The same variables were then tested in a backward multivariate Cox Regression analysis including baseline eGFR or CKD status to predict 28-day or 1-year mortality.

A p value <0.05 was considered statistically significant. All statistical analyses were performed using MedCalc software (version 12.0.4.0, MedCalc Software bvba, Mariakerke, Belgium).

## Results

### Study subjects

Between January 1st, 2007 and December 31st, 2009, a total of 1,368 patients were admitted to the medical ICU; 212 of these patients experienced septic shock as defined by the international consensus statement [8]. Forty-nine patients were excluded from the study because they were on chronic hemodialysis (n=21), had undergone kidney transplant (n=14) before their admission or lacked of baseline eGFR data (n=14).

One hundred sixty-three patients were analyzed, with 107 and 56 patients in the non-CKD and CKD groups, respectively. The median delay between the calculation of the baseline eGFR (considering the last stable serum creatinine value available) and the onset of septic shock was 22 days (3–73). In 32 non-CKD patients, the eGFR was determined after the recovery from the septic shock and in the absence of renal support. The delay between the end of the septic shock and the determination of the eGFR in this population of 32 patients was 9.5 days (3–18).

Patient characteristics are presented in Table 1. Age, serum creatinine on admission, serum creatinine at the onset of septic shock and SAPS II were significantly higher in CKD patients than in non-CKD patients. Similarly, the number of patients with a history of diabetes, heart failure, peripheral artery disease, coronary artery disease, and statin medication was greater in the CKD group than in the non-CKD group. The number of days without catecholamines was significantly lower in the CKD group. No significant inter-group differences were observed in terms of gender, Hb on admission, need for renal support, number of days without mechanical ventilation, length of stay and infection sites. Also the number of patients experiencing more than 1 septic shock during their ICU stay was similar between the 2 groups (2 (3.5%) CKD patients vs 9 (8.4%) non-CKD patients; p=0.34). The most frequent infection sites were the lungs (39%). As expected, baseline eGFR was significantly lower in the CKD group than in the non-CKD group (36 (28–44) vs. 81 (67–108) ml/min/1.73m^2^, respectively; p=0.001).

**Table 1:** Patient characteristics

**Parameter**	**Overall**	**Non-CKD**	**CKD**	**p value between Non-CKD and CKD**
	**n=163**	**n=107**	**n=56**	
Male gender	93 (57%)	58 (54%)	35 (62%)	0.4
Age (years)	66 (57–76)	63 (54–76)	72 (62–79)	<0.001
Diabetes	51 (31%)	20 (19%)	31 (55%)	0.001
HBP	97 (59%)	58 (54%)	39 (70%)	0.08
Heart failure	26 (16%)	7 (6%)	19 (34%)	0.001
Peripheral artery disease	25 (15%)	9 (8%)	16 (28%)	0.002
Coronary artery disease	28 (17%)	11 (10%)	17 (30%)	0.003
COPD	27 (17%)	18 (17%)	9 (16%)	0.9
Statin use	41 (25%)	20 (19%)	21 (37%)	0.01
Admission				
not related to surgery	145 (89%)	97 (91%)	48 (86%)	0.5
related to surgery	18 (11%)	10 (9%)	8 (14%)	0.4
ICU-acquired septic shock	11 (7%)	8 (7%)	3 (5%)	0.8
Baseline eGFR, (ml/min/1.73m^2^)	67 (44–92)	81 (67–108)	36 (28–44)	<0.001
Scr on admission (μmol/l)	241 (137–415)	187 (116–305)	400 (300–520)	<0.001
Scr onset of septic shock (μmol/l)	240 (131–390)	203 (102–332)	380 (236–469)	<0.001
Hb on admission (g/dl)	10.2 (8.7-12.1)	10.2 (8.6-12.2)	10.2 (8.9-11.8)	0.6
SAPS II	60 (45–78)	58 (43–69)	66 (55–85)	0.005
Days w/o catechol (days)	2 (0–9)	3 (0–9)	0 (0–5)	0.02
Days w/o MV (days)	1 (0–5)	2 (0–5)	1 (0–5)	0.9
Length of stay (days)	7 (3–14)	8 (3–15)	5 (2–13)	0.1
Infection sites				
lungs	64 (39%)	43 (40%)	21 (37%)	0.9
abdominal	25 (15%)	16 (15%)	9 (16%)	0.9
urinary	21 (13%)	12 (11%)	9 (16%)	0.5
cutaneous	12 (7%)	8 (7%)	4 (7%)	0.8
endocarditis	2 (1%)	0	2 (3%)	-
bone	2 (1%)	1 (1%)	1 (2%)	-
unknown	35 (21%)	25 (23%)	10 (20%)	0.5
RIFLE Classification	135 (83%)	86 (80%)	49 (87%)	0.4
Risk	25 (17%)	23 (21%)	2 (3%)	<0.001
Injury	26 (19%)	16 (15%)	10 (18%)	0.3
Failure	78 (58%)	47 (44%)	31 (63%)	0.08
Loss of function	6 (4%)	0	6 (12%)	-
ESKD	0	0	0	-
Renal support	75 (46%)	45 (42%)	30 (53%)	0.3
IHD	46 (33%)	29 (64%)	17 (57%)	0.9
CVVHDF	32 (23%)	19 (42%)	13 (43%)	0.6
CVVHF	16 (11%)	12 (27%)	4 (13%)	0.5
28-day mortality	93 (57%)	54 (50%)	39 (70%)	0.03
1-year mortality	115 (71%)	69 (64%)	46 (82%)	0.03

Reported as No. (%) or median (IQR).

*CKD* chronic kidney disease (Baseline eGFR <60ml/min/1.73m2), *Scr* serum creatinine, *BMI* body mass index, *HBP* High blood pressure, *ICU* intensive care unit, *SAPS II* Simplified Acute Physiologic Score II, *eGFR* estimated glomerular filtration rate, *adm.* admission, *MV* mechanical ventilation, *catechol.* catecholamines, *ESKD* end-stage kidney disease, *IHD* intermittent hemodialysis, *CVVHDF* continuous venovenous hemodiafiltration, *CVVHF* continuous venovenous hemofiltration.

**Figure 1:**
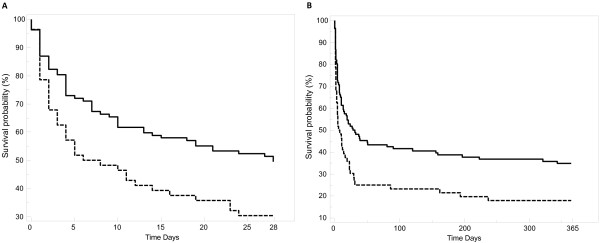
**Kaplan-Meier survival curves after (A) 28 days (log rank test: X**^**2**^**=6.86, p=0.009) and (B) 1 year (log rank test: X**^**2**^**=7.67, p=0.006).**

The mortality rate was significantly higher in the CKD group than in the non-CKD group after 28 days (70% vs. 50%, respectively; p=0.03) and after 1 year (82% vs. 64%, respectively; p=0.03). Twenty-eight-day and one-year survival curves are shown in Figure 1. The CKD and non-CKD curves differed significantly at both time-points but mostly during the first 28 days of hospitalization.

Compared with survivors, patients who died within 28 days of onset of septic shock were older and presented a higher SAPS II, a lower baseline eGFR, a lower number of days without mechanical ventilation and catecholamines and a longer length of stay (Table 2). However, no significant differences were observed for serum creatinine on admission or at the onset of septic shock, Hb on admission, gender, history of diabetes, hypertension, heart failure, peripheral artery disease, coronary artery disease, COPD or statin medication.

**Table 2:** Clinical and laboratory characteristics of survivors and non-survivors at 28 days

	**Survivors**	**Non-survivors**	**p**
	**n=70**	**n=93**	
Male gender	44 (63%)	49 (53%)	0.2
Age (years)	64 (53–74)	71 (59–77)	0.01
Diabetes	23 (33%)	28 (30%)	0.9
HBP	39 (56%)	58 (62%)	0.4
Heart failure	9 (13%)	17 (18%)	0.4
Peripheral artery disease	12 (17%)	13 (14%)	0.8
Coronary artery disease	11 (16%)	17 (18%)	0.8
COPD	15 (21%)	12 (13%)	0.2
Statin use	18 (26%)	23 (25%)	0.9
Baseline eGFR (ml/min/1.73m^2^)	78 (61–106)	63 (38–82)	0.001
CKD	17 (24%)	39 (42%)	0.03
Scr on admission (μmol/l)	209 (122–409)	290 (175–426)	0.15
Scr at onset of septic shock (μmol/l)	232 (117–384)	270 (174–396)	0.2
Hb on admission (g/dl)	10.5 (8.2-12.7)	10 (8.8-11.7)	0.4
SAPS II	50 (40–62)	68 (56–90)	<0.001
Days w/o catechol (days)	8 (3–14)	0 (0–2)	<0.001
Days w/o MV (days)	5 (2–8)	0 (0–1)	<0.001
Length of stay, days	12 (6–25)	4 (1–10)	<0.001
RIFLE Classification	59 (44%)	76 (56%)	0.08
Risk	14 (56%)	11 (44%)	0.7
Injury	14 (54%)	12 (46%)	0.8
Failure	27 (35%)	51 (65%)	0.009
Loss of function	4 (67%)	2 (33%)	0.7
ESKD	-	-	-

Reported as the number (%) or the median (IQR).

*Scr* serum creatinine, *CKD* chronic kidney disease (Baseline eGFR < 60ml/min/1.73m2), *BMI* body mass index, *HBP* High blood pressure, *SAPS II* Simplified Acute Physiologic Score II, *adm.* admission, *GFR* glomerular filtration rate, *MV* mechanical ventilation, *catechol.* catecholamines.

### Effect of baseline eGFR on survival: univariate and multivariate analyses

The results of univariate analysis for prediction of 28-day and 1 year mortality are presented in Table 3. Age, baseline eGFR, CKD and SAPS II were significant risk factors for 28-day mortality. The same variables and the history of heart failure were also significant risk factors for 1-year mortality.

**Table 3:** Univariate analysis of predictors of 28-day and 1-year mortality

	**28-day mortality**		**1-year mortality**	
	**HR (95% CI)**	**p**	**HR (95% CI)**	**p**
Male gender	1.25 (0.84-1.88)	0.27	1.02 (0.71-1.48)	0.89
Age	1.02 (1.01-1.04)	0.001	1.02 (1.01-1.04)	0.002
Diabetes	1.01 (0.65-1.58)	0.95	1.13 (0.76-1.66)	0.55
Hypertension	1.36 (0.89-2.07)	0.15	1.33 (0.91-1.93)	0.14
Heart failure	1.52 (0.90-2.57)	0.12	1.81 (1.19-2.75)	0.005
Peripheral artery disease	0.89 (0.50-1.60)	0.71	0.7 (0.54-1.51)	0.9
Coronary artery disease	1.20 (0.71-2.02)	0.5	1.08 (0.67-1.75)	0.74
COPD	0.69 (0.38-1.26)	0.23	0.93 (0.58-1.51)	0.78
Statin use	1.08 (0.68-1.73)	0.73	1.14 (0.75-1.73)	0.55
Baseline eGFR	0.99 (0.98-0.99)	0.006	0.99 (0.98-0.99)	0.004
CKD	1.69 (1.13-2.56)	0.01	1.67 (1.15-2.42)	0.007
Scr on admission	1.01 (0.99-1.01)	0.67	1.01 (0.99-1.01)	0.23
Scr at septic shock onset	1.02 (0.99-1.01)	0.71	1.01 (0.99-1.01)	0.26
Hb on admission	0.95 (0.88-1.03)	0.23	0.95 (0.88-1.01)	0.12
SAPS II	1.04 (1.03-1.05)	0.001	1.04 (1.03-1.05)	0.001

*CI* confidence interval, *SAPS II* Simplified Acute Physiologic Score II, *GFR* glomerular filtration rate, *CKD* chronic kidney disease (Baseline eGFR <60ml/min/1.73m^2^); *Scr* serum creatinine.

Multivariate Cox regression analysis demonstrated that baseline eGFR or CKD status were independent predictive factors of 28-day mortality after adjustments for multiple confounders (age, gender, history of diabetes, hypertension, heart failure, peripheral artery disease, coronary artery disease, COPD, statins, creatinine and Hb on admission) (Table 4). CKD status (but not baseline eGFR) remained an independent predictor of 28-day and 1-year mortality when SAPS II was added to the previous list of confounders (Tables 4 and 5).

**Table 4:** Multivariate Cox regression analysis of baseline risk factors for 28-day mortality

	**Hazard ratio**	**95% CI**	**p**
Model 1*			
Age	1.02	1.01-1.03	0.04
Baseline eGFR	0.99	0.98-0.99	0.05
Model 2**			
Age	1.02	1.00-1.04	0.01
CKD	1.54	1.01-2.35	0.04
Model 1 + SAPS II			
SAPS II	1.04	1.03-1.05	0.001
Model 2 + SAPS II			
Peripheral artery disease	0.48	0.25-0.93	0.03
CKD	1.7	1.08-2.68	0.02
SAPS II	1.04	1.03-1.05	0.001

*, Model 1 includes: Age, Gender, History of Diabetes, Hypertension, Heart failure, Peripheral artery disease, Coronary artery disease, COPD, Statins, Creatinine on admission, Hemoglobin on admission, Baseline eGFR.

**, Model 2 includes: Age, Gender, History of Diabetes, Hypertension, Heart failure, Peripheral artery disease, Coronary artery disease, COPD, Statins, Creatinine on admission, Hemoglobin on admission, CKD (Baseline eGFR <60ml/min/1.73m^2^).

*CI* confidence interval, *CKD* chronic kidney disease, *eGFR* glomerular filtration rate.

**Table 5:** Multivariate Cox regression analysis of baseline risk factors for 1-year mortality

	**Hazard ratio**	**95% CI**	**p**
Model 1*			
Age	1.02	1.01-1.03	0.01
Heart failure	1.83	1.18-2.83	0.007
Hemoglobin on admission	0.91	0.85-0.98	0.01
Model 2**			
Age	1.02	1.00-1.04	0.004
Peripheral artery disease	0.56	0.32-0.98	0.04
Hemoglobin at admission	0.92	0.85-0.99	0.02
CKD	1.62	1.09-2.40	0.02
Model 1 + SAPS II			
Heart failure	2.25	1.29-3.93	0.005
Coronary artery disease	0.40	0.21-0.77	0.006
Hemoglobin on admission	0.91	0.84-0.98	0.009
SAPS II	1.04	1.03-1.05	0.001
Model 2 + SAPS II			
Heart failure	1.70	0.99-2.88	0.05
Peripheral artery disease	0.51	0.28-0.95	0.03
Coronary artery disease	0.49	0.26-0.93	0.03
Hemoglobin on admission	0.91	0.84-0.98	0.01
CKD	1.67	1.08-2.57	0.02
SAPS II	1.04	1.03-1.05	0.001

*, Model 1 includes: Age, Gender, History of Diabetes, Hypertension, Heart failure, Peripheral artery disease, Coronary artery disease, COPD, Statins, Creatinine on admission, Hemoglobin on admission, Baseline eGFR.

**, Model 2 include: Age, Gender, History of Diabetes, Hypertension, Heart failure, Peripheral artery disease, Coronary artery disease, COPD, Statins, Creatinine on admission, Hemoglobin on admission, CKD (Baseline eGFR <60ml/min/1.73m^2^).

*CI* confidence interval, *CKD* chronic kidney disease, *eGFR* glomerular filtration rate.

## Discussion

In this study, non-dialysis CKD and baseline eGFR were found to be independent risk factors for death in septic shock patients. The leading cause of death in CKD patients is cardiovascular disease, but it accounts for only 50% of overall mortality. The causes of the remaining 50% mortality have been less intensively studied. The second leading cause of mortality in end-stage kidney disease patients, after cardiovascular disease, is infectious disease. Previous studies have clearly established CKD as a strong risk factor for septicemia and lung infection [11-13]. However, the prognosis of infections in non-dialysis CKD patients has been less well studied, despite the fact that the incidence of infectious disease and the associated mortality rate in non-dialysis CKD patients are known to be higher than in non-CKD populations [14]. Adult patients with bacteremia and serum creatinine on admission >266 μmol/l are at increased risk of death (although creatinine on admission does not exactly reflect baseline GFR) [15]. In our study, serum creatinine on admission was elevated but similar in both survivors and non-survivors. This result suggests that serum creatinine on admission is mainly the result of septic-shock-related acute (but possibly reversible) kidney damage and does not have the same prognostic impact as chronic changes in baseline eGFR. James *et al.* demonstrated that non-dialysis CKD patients are exposed to a higher risk of lung infection and a higher risk of death from lung infection [7]. They also observed an increased risk of bloodstream infections and death from community-acquired bloodstream infections in non-dialysis CKD patients [6]. However, the study by James *et al.* dealt with infections in general and did not specifically focus on septic shock.

The mortality rate in our population of septic shock patients (57% after 28 days) was similar to the usually reported values of 40% to 70% [16,17]. Moreover, the SAPSII-predicted hospital mortality of our population was about 70%, which is fairly similar to the observed rate of 65% [18]. In an observational cohort of more than 192,000 patients in the United States, Angus *et al.* reported an increased mortality rate from severe sepsis in CKD patients (36.7%), relative to the overall study population (28.6%) [1]. However, this study concerned sepsis rather than septic shock in particular and CKD was not clearly defined. Similarly, Alberti *et al.* reported an increased frequency of CKD in non-survivors from a cohort of 3,608 infected ICU patients [19]. Annane *et al.* looked for prognostic factors in a population of 8,250 septic shock patients in Europe and North America [16]. As in the study by Alberti et al., CKD was not reported as an independent risk factor for death in septic shock. However, Annane *et al.* did not clearly defined CKD and did not specify whether chronically dialyzed patients and kidney transplant patients were included in the analyses. The strengths of our study are that it focuses on septic shock (i.e. the most lethal infectious state) with CKD defined according to international guidelines (i.e. as a function of baseline GFR) [20].

In 14 patients we could not determine the eGFR in the absence of stable serum creatinine before the septic shock and the absence or abnormal eGFR after the episode. A proportion of those patients could be unknown CKD patients or non-CKD patients suffering of chronic kidney failure after recovering from the septic shock. Among those 14 patients, 9 (64%) died (all during the first 28 days after the onset of septic shock). Even if we hypothesized that those 14 patients all belonged to the non-CKD group this would have only slightly modified the 28 day mortality rate (from 50% to 52%) and the difference with the CKD mortality rate would remained significant (p=0.033). It is also usually reported that RIFLE classification (defined as an increase of creatinine above the baseline creatinine value) is associated with mortality in septic shock patients [21]. In our study the degree of AKI (RIFLE classification) was not predictive of mortality. Although RIFLE is not a significant risk factor in the uni and multivariate analyses in our study, we still find a significant difference between survivors and non-survivors for the RIFLE Failure groups and close to significant for the global RIFLE classification (p=0.08) (Table 2). This partial result could be the consequence of an insufficient number of patients. However this result could also result from a reduced increase of creatinine during sepsis as suggested by a study in septic mice by Doi et al. [22]. The authors showed a decreased production of creatinine during sepsis in mice and in our study this could have reduced the elevation of creatinine, altering the RIFLE classification.

The introduction of SAPS II into the multivariate models modified the relation between the different variables and the mortality. Generally speaking, the multivariate backward analyze introduces all the variables into the model and than delete the variable that will improves the most the predictive model by being deleted. This process is repeated until no further improvement is possible. In our study, the age dropped below the significance threshold (Tables 4 and 5) when including SAPS II in the different models meaning that the elimination of the age improved the prediction of mortality. The reason is that SAPS II is a predictive hospital mortality score combining 17 different variables including the age. This explains the stronger predictive value of SAPS II than the age alone in our models.

Also the introduction of SAPS II raised the history of peripheral artery disease (Model 2+SAPS II Table 4) and coronary artery disease (Model 1 and 2+SAPS II Table 5) above the significance threshold. This signifies that those 2 histories bring additional values to predict the mortality that are not shared with SAPS II but were with the age.

The mechanisms linking CKD to increased mortality during sepsis have not been fully elucidated, but hemodynamic and immune causes may be involved. We can hypothesize a possible role of the cardiovascular alterations linked to CKD that could aggravate septic shock. The development of left ventricular hypertrophy, diastolic dysfunction and/or aortic stiffness related to even early stage of CKD is associated with higher cardiovascular morbidity and mortality [4]. In the context of septic hemodynamic stress, these abnormalities could be accentuated, resulting in increased mortality. However, in the present study, CKD appeared to be a risk factor independent of cardiovascular comorbidities. Kidney failure and uremia are associated with severe alterations in the immune system. The two mechanisms required for complete activation of T cells are compromised [23,24]. After activation, T lymphocytes differentiate into Th1 or Th2 lymphocytes that promote cell-mediated and humoral immunity respectively. This differentiation is depressed in uremic patients [25]. Moreover, in CKD, the neutrophil count is not altered but neutrophils are less able to kill microorganisms after phagocytosis [26].

Our findings in humans are supported by data from two rodent models. Sepsis (induced by cecal ligature-puncture) in mice with pre-existing CKD (created by 5/6th nephrectomy or the injection of folic acid) was associated with a higher mortality rate than in septic non-CKD mice [27,28]. The presence of kidney dysfunction prior to sepsis was associated with increased vascular permeability, bacteremia, elevated vascular endothelial growth factor and serum IL-10 levels and splenocyte apoptosis. More recently, the increased release of high-mobility group box protein 1 (HMGB1) and splenic apoptosis in septic CKD mice has highlighted these pathways in the CKD/sepsis interaction [28].

Our study is limited by its small sample size preventing analysis of the effect of an eGFR between 30 and 59 ml/min/1.73m^2^ (stage 3 CKD) or between 15 and 29 ml/min/1.73m^2^ (stage 4 CKD) on risk factors in this population, which could only be divided into two groups (eGFR ≥ or < 60ml/Kg/1.73m^2^). It is also important to note the high prevalence of CKD in our population of patients hospitalized for septic shock in our medical ICU (43% when taking into account the excluded transplanted and chronically hemodialysis patients). As a retrospective single-center study performed in a Nephrology Department’s medical ICU, patient recruitment was probably biased towards CKD.

## Conclusions

Non-dialysis CKD appears to be an independent risk factor for death after septic shock in ICU patients. These results emphasize the importance of kidney function before onset of septic shock. Although the mechanisms underlying this elevated mortality rate have yet to be explored, severe alterations of the cardiovascular and/or immune systems may well play a major role.

## Abbreviations

CKD: Chronic kidney disease; COPD: Chronic obstructive pulmonary disease; eGFR: Estimated glomerular filtration rate; ICU: Intensive care unit; SAPS II: Simplified Acute Physiologic Score II; MV: Mechanical ventilation; Scr: Serum creatinine; Hb: Hemoglobin; ESKD: End-stage kidney disease; IHD: Intermittent hemodialysis; CVVHDF: Continuous venovenous hemodiafiltration; CVVHF: Continuous venovenous hemofiltration

## Competing interests

The authors declared that they have no competing interests.

## Authors’ contributions

JM and RD ensured data acquisition and wrote the manuscript. JM, RD and ZM performed statistical analysis. BD, ES, EZ were involved in conception, design and data acquisition. JM, CT, ZM, GC and MS were involved in the conception and coordination of the study and corrected the manuscript. All authors have read and approved the final manuscript.

## Pre-publication history

The pre-publication history for this paper can be accessed here:

http://www.biomedcentral.com/1471-2369/14/77/prepub
